# Building Interdisciplinary Partnerships for Community-Engaged Environmental Health Research in Appalachian Virginia

**DOI:** 10.3390/ijerph17051695

**Published:** 2020-03-05

**Authors:** Emily Satterwhite, Shannon Elizabeth Bell, Linsey C. Marr, Christopher K. Thompson, Aaron J. Prussin, Lauren Buttling, Jin Pan, Julia M. Gohlke

**Affiliations:** 1 Appalachian Studies, Department of Religion and Culture, Virginia Tech, Blacksburg, VA 24061, USA; 2 Department of Sociology, Virginia Tech, Blacksburg, VA 24061, USA; 3 Department of Civil and Environmental Engineering, Virginia Tech, Blacksburg, VA 24061, USA; lmarr@vt.edu (L.C.M.); Laurenb3@vt.edu (L.B.); jinpan@vt.edu (J.P.); 4 School of Neuroscience, Virginia Tech, Blacksburg, VA 24061, USA; ckt@vt.edu; 5 Department of Population Health Sciences, Virginia Tech, Blacksburg, VA 24061, USA; jgohlke@vt.edu

**Keywords:** environmental health, interdisciplinary research, transdisciplinary research, community-engaged research, Appalachia

## Abstract

This article describes a collaboration among a group of university faculty, undergraduate students, local governments, local residents, and U.S. Army staff to address long-standing concerns about the environmental health effects of an Army ammunition plant. The authors describe community-responsive scientific pilot studies that examined potential environmental contamination and a related undergraduate research course that documented residents’ concerns, contextualized those concerns, and developed recommendations. We make a case for the value of resource-intensive university–community partnerships that promote the production of knowledge through collaborations across disciplinary paradigms (natural/physical sciences, social sciences, health sciences, and humanities) in response to questions raised by local residents. Our experience also suggests that enacting this type of research through a university class may help promote researchers’ adoption of “epistemological pluralism”, and thereby facilitate the movement of a study from being “multidisciplinary” to “transdisciplinary”.

## 1. Introduction

In recent decades, there have been increasing calls for academics to move beyond disciplinary silos to engage in transdisciplinary research aimed at addressing the “wicked problems” [1] faced by society, such as climate change, poverty, political instability, and complex environmental health problems, such as air and water pollution [2]. There is a recognition that addressing such challenges requires the expertise of scholars from diverse disciplines and epistemological orientations [3]. There is, however, little acknowledgment of the difficulties inherent in collaborating across these differences. Indeed, achieving transdisciplinary resolutions are difficult precisely *because* of the different epistemological orientations of various fields [4].

Miller et al. 2008 argue that achieving research that is truly transdisciplinary requires a commitment to “epistemological pluralism”, or a recognition that, “in any given research context, there may be several valuable ways of knowing, and that accommodating this plurality can lead to more successful integrated study” (p. 46, [4]). However, recognizing and unlearning our disciplinary biases can be a challenge, and attempts at transdisciplinary research can be easily thwarted by a lack of attention to understanding these differences and how they can strengthen the research design. 

Here we suggest that one approach to moving community-responsive research from a multidisciplinary project toward a transdisciplinary collaboration is through designing the study to be part of a community-based interdisciplinary research class. Working as an interdisciplinary team on the curriculum provides the opportunity for team members to learn more about each other’s epistemologies. Figuring out how to articulate to the students how the researchers’ differing methods achieve different goals for the research project is not only clarifying for the students, but it also requires self-reflection and openness to alternative ways of knowing for the researchers involved in the project. 

In the sections that follow, we describe a collaboration among an interdisciplinary group of university faculty who sought to address long-standing community concerns about the environmental health effects of the U.S. Army’s Radford Army Ammunition Plant in southwest Virginia. The research was conceptualized, designed, and conducted in conjunction with an undergraduate research course that engaged multiple stakeholders. Two local county Boards of Supervisors agreed to serve as formal partners, thanks in part to Army staff endorsement of the project. Local residents both prompted the research questions and participated as survey respondents and interviewees. Enacting this research through a university class helped promote epistemological pluralism among our team members while also providing an effective site for integrating, synthesizing, and sharing the research. Enabling students to respond to a community need served as the foundation on which our different epistemological perspectives converged, and the class was the mechanism that facilitated the process of moving from conducting multidisciplinary projects to being part of a transdisciplinary study. We maintain that because scholars in different disciplines operate based on differing incentives, research costs, and teaching duties, the academy’s ability to be responsive to public needs requires deliberate institutional practices to facilitate and incentivize transdisciplinary research through providing resources and structures, such as the ability to co-teach small, interdisciplinary classes. 

## 2. Materials and Methods 

### 2.1. Requisite Conditions

This research emerged from a confluence of community advocacy focused on environmental health concerns, openness to research collaborations on the part of leadership at the Radford Army Ammunition Plant, university funding for and structural encouragement of transdisciplinary work, and faculty members’ willingness to engage other disciplines and adopt research agendas shaped by contentious local demands. In the sections below, we describe these essential conditions, which made this research possible.

#### 2.1.1. Community Environmental Health Advocacy

**Figure 1:**
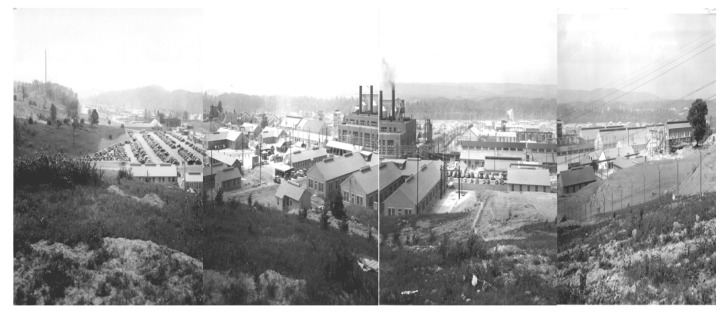
Radford Ordnance Works (Radford Army Ammunition Plant), 1941. [11].

**Figure 2:**
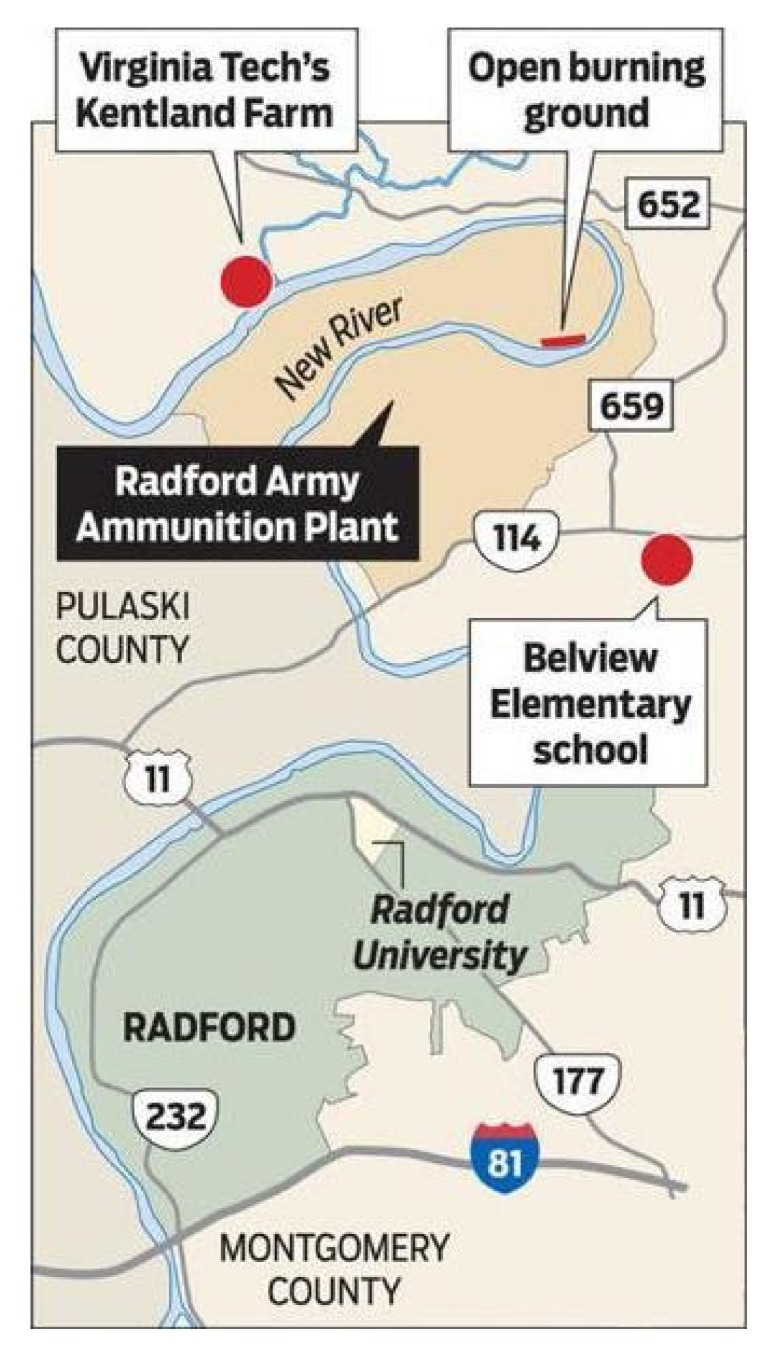
Map of the Radford Army Ammunition Plant and Surrounding Area. Image reproduced courtesy of the *Richmond Times-Dispatch* [12].

**Figure 3:**
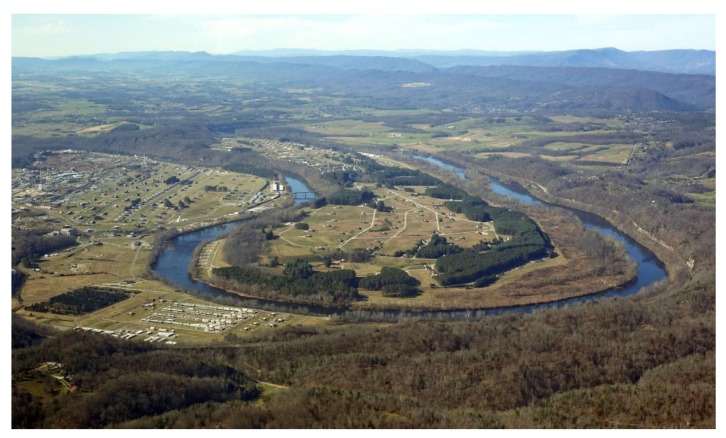
Aerial Image of Radford Army Ammunition Plant, 2006. Photo by Steve DeHart.

The Radford Army Ammunition Plant (RAAP) has been Virginia’s largest polluter since 2001 [5]. Well-known environmental activist and whistleblower Erin Brockovich has referred to RAAP as “One of the most dangerous and toxic facilities in the Nation” [6]. Established in 1941 in the New River Valley of southwest Virginia, the Radford Army Ammunition Plant employed 23,000 people during the height of World War II (Figure 1). In 2017, “the Arsenal”, as it is widely known, still employed approximately 2500 people in the manufacture of bullets, rockets and other munitions, making it the eighth largest employer in the region [7]. While community loyalty to this major employer has been fierce among multigenerational community members, other residents have increasingly expressed concerns about the facility’s impact on environmental health and have demanded data and action regarding emissions. Of particular concern to these residents has been RAAP’s “open burn” site at the edge of the New River, where manufacturing waste that is not compatible with the incinerator is burned in the open air as a method of disposal [7,8] (Figure 2 and Figure 3). 

During the 2010s, community activism focused on RAAP escalated. Multiple watchdog groups formed in order to insist upon greater transparency regarding the production, disposal, and effects of pollutants at RAAP, including Environmental Patriots of the New River Valley (2015–2019), Hold Radford Arsenal Accountable (2015–present), and Citizens for Arsenal Accountability (2017–2018). In 2017, coverage of the US military’s open burn sites by *ProPublica* [9] led to a surge of renewed interest and concern surrounding the public health effects of the Radford Arsenal. The town council of nearby Blacksburg, Virginia, passed a resolution “Urging the Radford Army Ammunition Plant to Address Environmental Hazards,” and requested data on the effects of the facility’s emissions on air, water, and soil [10]. 

#### 2.1.2. RAAP Responsiveness

RAAP had signaled responsiveness to community concerns dating at least to the formation of a Restoration Advisory Board in 1998. In 2012, RAAP began holding quarterly meetings with the public. Community concerns also led to the siting of a Virginia Department of Environmental Quality (DEQ) air monitor at a nearby location. However, the site (a wastewater treatment plant) was in the opposite direction from prevailing winds, so the reported low levels of emissions were not necessarily reassuring for residents more directly downwind of the open burn site. 

Under the command of Lieutenant colonel Alicia Masson from 2015 to 2017, RAAP pledged greater transparency, increased the number of public meetings from two to five per year, initiated plans for a new incinerator that could handle more of the waste that had been going to the open burning grounds, and oversaw the closure of the coal plant [13]. Further, during Masson’s tenure, the Army commissioned a drone study to characterize air pollutant emissions produced by open burning at RAAP [14,15], an indication of openness to new research collaborations focused on addressing community demands. 

In 2017, in response to the community’s concerns reflected in the Blacksburg Town Council’s resolution, Commander Lt. Col. James Scott proposed meeting with local officials to address inaccuracies [16] and invited the local health department to present data on thyroid cancer incidence at a quarterly meeting. It was during this period of RAAP’s increased efforts to respond to community demands that faculty at nearby Virginia Tech reached out to inquire about RAAP’s interest in supporting a research project done in collaboration with an undergraduate course, and RAAP staff swiftly welcomed the opportunity. 

#### 2.1.3. University and Faculty Support of Transdisciplinary Community-Engaged Research

At the same time that RAAP was increasing efforts at transparency in response to community advocacy, an interdisciplinary group of faculty at Virginia Tech had begun to collaborate around issues of environmental health in rural Appalachia. In 2016, the faculty group coalesced around the topic of ecological and human health in rural communities (henceforth referred to as the Rural Environments Group) in order to engage with a new Virginia Tech Global Systems Science initiative to tackle complex topics by building transdisciplinary teams that would undertake teaching, research, and engagement activities. Faculty involved in the Rural Environments Group were from a variety of disciplines, including Population Health Sciences, Appalachian Studies, Biological Systems Engineering, Civil and Environmental Engineering, Geography, Neuroscience, Sociology, Horticulture, Veterinary Medicine, Natural Resources, Statistics, and Science and Technology Studies, among others. In 2017, the group decided to initiate an interdisciplinary, community-engaged research project on possible environmental health hazards related to RAAP. 

The aforementioned drone study commissioned by RAAP provided data that allowed Virginia Tech researchers to establish whether there was a reasonable cause for environmental health concerns. Researchers with NASA, the Environmental Protection Agency, and the University of Dayton Research Institute piloted an unmanned aerial vehicle (UAV) equipped to measure gases and particles through 25 plumes over two weeks of sampling [14,15]. The UAV measured carbon dioxide, carbon monoxide, and fine particulate matter smaller than 2.5 μm (PM_2.5_) in real time and collected gas and particle samples that were analyzed for 61 volatile organic compounds (VOCs), nitroaromatics, nitrocellulose, hydrochloric acid, perchlorate, chlorate, dioxins, furans, and metals. At high enough levels of exposure, some of these compounds may present a risk to human health. The results of the study demonstrated that emission factors of PM_2.5_ and individual metals other than lead were comparable to those for combustion of refuse under uncontrolled (i.e., no pollution control equipment) conditions [17]. Emission factors of dioxins and furans were comparable to those from forest burns [15]. Nitroaromatics, nitrocellulose, chlorate, perchlorate, mercury, and many of the VOCs were not detected in the plumes. 

The emission factor that stood out most to researchers in the Rural Environments Group was that for lead, a metal with well-known neurological effects. It was five times higher than expected and implied that burning a typical amount of waste on one day would release 14 kg of lead into the atmosphere. However, a limitation of the study was that lead’s emission factor could not readily be translated into an estimate of risk because lead would be dispersed in the atmosphere such that concentrations downwind would be greatly diluted compared to those measured in the plume.

There was a seeming disconnect between the results of the study and the community’s primary environmental health concerns, as residents’ concerns had most often centered on thyroid cancer, not the neurotoxic effects for which lead is best known. However, a neuroscientist in the Rural Environments Group noted that lead poisoning is, in fact, associated with changes in thyroid function, although a specific connection between lead toxicity and thyroid cancer has not been conclusively demonstrated. Lead poisoning is associated with a decrease in circulating levels of thyroid hormone distributor proteins in lab animals [18,19] and in the cerebral spinal fluid of humans [20], suggesting this as a possible mechanism by which lead poisoning may lead to an overgrowth of the thyroid gland. This insight contributed to the Rural Environments Group’s determination that further investigations into the lead emissions from open burning at RAAP were warranted. 

The group decided that pursuing community-based research through an undergraduate “Appalachian Community Research” course could be a fruitful way to meet the research, teaching, and community-engagement objectives of the Rural Environments Group. This interdisciplinary class, which is taught each fall, regularly receives funding from the Appalachian Regional Commission’s Appalachian Teaching Project to involve students in conducting community-engaged research on issues related to building a sustainable future for Appalachian communities. It was also decided that Rural Environments faculty would conduct three pilot studies during the Spring and Summer to provide findings that could help inform the community-based research undertaken by the students in the Appalachian Community Research class the following Fall. 

### 2.2. Pilot Study Methods

The three pilot studies entailed collecting and testing air, soil, and biological samples downwind of RAAP as an indicator of current and historic levels of lead in the environment. Gas and particle samples were collected on site at RAAP and at two nearby residential sites. These samples were analyzed for five chemicals that are listed by the US Environmental Protection Agency as hazardous air pollutants (benzene, toluene, ethylbenzene, meta-xylene, para-xylene, and ortho-xylene) and six metals, including arsenic (a metalloid), cadmium, cobalt, copper, nickel, and lead. The gas samples were extracted in carbon disulfide and analyzed by gas chromatography with mass spectrometry. The metals in particle samples were dissolved by microwave acid digestion and analyzed by inductively coupled plasma mass spectrometry (ICP–MS). Quality assurance and quality control measures included the collection and analysis of field blanks, laboratory blanks, reagent blanks, and the use of standard calibration curves. 

Twenty-nine soil samples were collected in areas generally downwind of RAAP, using concentric circles that were 1, 2, 3, 4, 5, 7, 10, and 12 km from the RAAP burn site. Soil samples were also taken from nearby Kentland Farm and at a distant control site. Soil samples were digested by the Soils and Landscape Rehabilitation Laboratory in the School of Plant and Environmental Sciences, and digests were analyzed by the Department of Crop and Soil Environmental Sciences using ICP–MS. 

Finally, Virginia Tech’s research cows at Kentland Farm (directly across the New River from RAAP) were used as a sentinel species to measure blood levels of lead and thyroid hormone distributor proteins. Blood from 15 cows was collected in April 2018. Plasma was transferred to the Virginia-Maryland College of Veterinary Medicine laboratory services for analysis.

### 2.3. Appalachian Community Research Course Methods

While community interest in the topic was well documented, requirements for the Appalachian Regional Commission’s grant also included the active participation of locally based organizations as partners who help define the needs and goals of the research project undertaken by students. Rural Environments faculty and RAAP staff agreed that the Boards of Supervisors for Pulaski and Montgomery Counties (the counties in which the Arsenal property is located) should be the community partners rather than RAAP so that the public would be more likely to trust the research findings. The Supervisors were somewhat hesitant, as neither Board wanted to appear adversarial towards a major employer. Past relationships with the Appalachian Studies program at Virginia Tech and especially RAAP’s support of the research were critical for securing the Boards’ participation. 

In all, approximately eight RAAP staff, six faculty members, and one Supervisor from each county participated in the initial collaboration meeting in February 2018. All parties agreed on the scope of the research: students enrolled in the Fall 2018 Appalachian Community Research course would identify and summarize existing relevant studies and data; seek out and catalogue residents’ concerns about the Radford Arsenal; and, if deemed advisable based on pilot studies, conduct additional air sampling for carcinogenic organic compounds and possibly lead. 

Cross-disciplinary conversations and negotiations among faculty with backgrounds in Appalachian Studies, environmental health, and sociology led to the syllabus for the Appalachian Community Research class. Faculty also discussed the methods that the students would use to catalogue residents’ concerns about the Radford Arsenal. They decided students would develop an online survey that would be distributed in the New River Valley and would also conduct interviews with a sample of local residents. Both surveys and interviews were deemed important to this study because of the different purposes these methods serve. The online survey could be distributed to a large sample in the region and would allow the students to capture a snapshot of people’s perceptions of the Arsenal and be able to analyze the role that employment plays in risk perception. The survey would also allow students to see which concerns were most relevant among the respondents. Interviews would provide additional nuance and specificity to the survey responses, allowing the students to learn more about the history of residents’ concerns, whether those concerns were based on specific observations or experiences, and what community members thought the Arsenal should do to address those concerns. 

The nine students that completed the Appalachian Community Research course brought a range of interests and skills to the table. Their majors ranged from biochemistry to human development, political science to environmental policy and planning, and sociology to environmental science. All students were required to complete human subjects research training provided by the Virginia Tech Institutional Review Board (IRB) so that they could conduct the proposed research. Students worked in pairs to conduct interviews. An important feature of the course was the provision of a teaching assistant in the form of Appalachian Transition Fellow, whose position was funded in part by Highlander Research and Education Center, thanks to the availability of matching funds from the Rural Environments Group. Students also benefited from in-lab instruction in air monitoring devices and in-class guest lectures by researchers involved in the pilot study data collection and analysis. One of the students’ first requests was to see the RAAP facility for themselves, and RAAP staff quickly facilitated a tour. 

Students began the course with readings and discussions with local officials to gain an understanding of the role RAAP plays in the New River Valley. They performed additional research independently in order to develop a review of the literature on environmental justice, the history of RAAP, existing regulatory frameworks, and public attitudes and concerns about RAAP’s effects on human well-being relative to extant knowledge regarding water, soil, and air quality. 

Students worked with faculty instructors and a county supervisor to develop the online survey to gather views on the role of the Arsenal in community members’ lives and any environmental or health concerns that respondents felt needed to be addressed. The survey was reviewed by local officials and Army staff and then distributed via email invitation with a link to the survey to Pulaski County and Montgomery County employees, Pulaski County school employees, Arsenal community meeting registrants, and Arsenal employees, as well as to the Facebook pages of two Arsenal watchdog groups. 

In order to provide more depth to the data collected through the survey, students also conducted semi-structured interviews. Triangulating methods in this way allowed us to capture a more complete and nuanced picture of community perceptions and concerns about the Arsenal. Students were trained on interviewing methodology, including question type and wording, common pitfalls in question formulation, question sequencing, techniques for building rapport, active listening skills, and approaches to interview transcript coding and analysis. The students assisted in developing the interview question protocol and practiced their interviewing techniques by pretesting the interview questions with each other before contacting potential interview participants. 

Students recruited 13 interviewees for this research in three ways: contacting survey respondents who indicated they were interested in being interviewed, inviting attendees at RAAP’s quarterly meetings, and contacting references made by a member of the Pulaski County Board of Supervisors. Students conducted the interviews in pairs and transcribed the interviews from audio recordings. Interview transcripts were analyzed with four themes in mind: benefits of the Arsenal, concerns and frustrations with the Arsenal, observed changes over time, and recommendations for improvements the Arsenal could make. 

The total cost of the various components of the project was $35,810. Two of the pilot studies were completed using seed funding from the Rural Environments Group: $5637 for the cow blood study and $4673 for the soil study. An additional $15,000 of discretionary research funds and in-kind support for the air study were provided by one of the faculty members in the Rural Environments Group. The course was supported by $4500 funding from the Appalachian Regional Commission—most of which went toward student travel to the ARC conference, but a few hundred of which supported student transportation for meetings and field trips within the affected communities. The course also benefitted from $6000 from the Rural Environments Group, which helped fund a teaching assistant.

## 3. Results

### 3.1. The Pilot Studies

**Figure 4:**
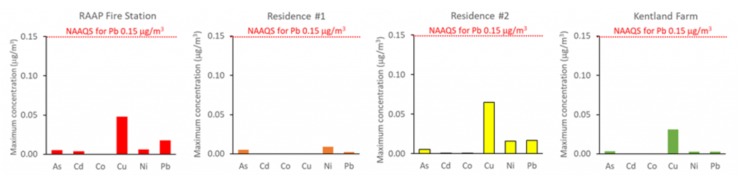
Maximum observed airborne concentrations of arsenic (As), cadmium (Cd), cobalt (Co), copper (Cu), nickel (Ni), and lead (Pb). Observed levels of all five elements were well below 0.15 μg/m^3^ (indicated by a red line), which is the standard for lead, which has the most stringent air quality or workplace standard of the five elements.

**Figure 5:**
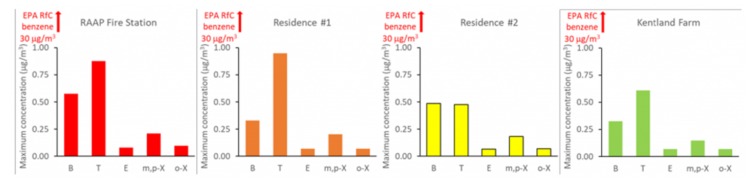
Maximum observed airborne concentrations of benzene (B), toluene (T), ethylbenzene (E), meta- and para-xylene (m,p-X), and ortho-xylene (o-X). Observed levels of all five volatile organic compounds were well below 30 μg/m^3^ (indicated by an arrow on each graph that points off the chart range), which is the standard for benzene, the most stringent Reference Concentration (RfC), as set by the Environmental Protection Agency (EPA), of the five volatile organic compounds (VOCs).

The concentrations of all air pollutants measured at RAAP and nearby sites were well below health-based standards for these compounds (Figure 4 and Figure 5).

**Figure 6:**
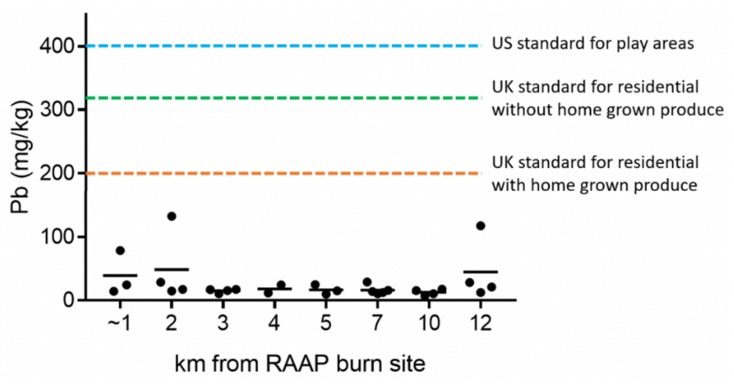
Graph comparing the amount of lead found in each soil collection site—each of which are separated by its distance from the open burning grounds. The most noticeable points are a site 1 km away that had approximately 80 mg/kg lead, a site from 12 km away with approximately 125 mg/kg lead, and the highest amount of lead, approximately 140 mg/kg, found 2 km away. RAAP—Radford Army Ammunition Plant.

Average soil lead levels at Kentland Farm were 12.1 mg/kg (*n* = 3). Soil samples throughout the area ranged from 7.3 to 132.5 mg/kg, with an average soil lead level of 26.6 mg/kg across the 29 sites east of RAAP. All measured levels were well below 400 mg/kg, the level the Environmental Protection Agency (EPA) considers to be minimum for remediation for soils in children’s play areas (Figure 6). Arsenic and cadmium levels were also well below the strictest standards available.

Blood lead levels from 13 cows were below the level of sensitivity (2.0 µg/dL), and only two cows had detectable blood lead levels (2.1 and 2.8 µg/dL). The EPA’s standard level of concern in children is 5.0 µg/dL. The data show that blood lead levels in the Kentland Farm cows are well below what would be of concern in humans. Given that blood lead levels were undetectable in the majority of cows, thyroid hormone distributor proteins were not analyzed.

The preliminary studies offered no support for the hypothesis that RAAP burns have polluted the surrounding area with lead. Within the limitations of the studies, these findings suggest that lead from the burn plume was dispersed widely enough that relatively small amounts of lead, not detectable above the background levels, remained in the air or settled onto soil nearby. 

### 3.2. The Appalachian Community Research Course Findings

#### 3.2.1. Survey Results

A total of 454 survey responses were received, with 61% from Pulaski County, 31% from Montgomery County, and 8% from the City of Radford. Of the respondents, 40% identified as female, 93% identified their ancestry as white or European, 3% as Black or African, 3% as American Indian or Alaska Native, and 1% as Hispanic or Latino. In addition, 55% reported earning above $75,000 each year and 64% reported earning a bachelor’s degree or higher. Reported income and level of education of the respondents are both higher than the demographics of the local area, based on the most recent census (19% bachelor’s degree or higher and median household income of $49,691 in Pulaski County and 46% bachelor’s degree or higher and median household income of $53,424 in Montgomery County); thus, these results are not generalizable, as they represent a more educated and higher-income demographic. 

Forty-six percent of respondents reported that either they or a household member worked for the Arsenal. Responses to the question “Do you have any concerns related to the Arsenal?” reflected less concern from respondents who had a household work history with the Arsenal (69% choosing probably or definitely not) versus respondents without a household work history with the Arsenal (38% choosing probably or definitely not). While the statement “Overall, I believe the arsenal is a positive presence in the New River Valley” best described the general feelings of 47% of respondents without a household work history with the Arsenal, it best described 89% of respondents with a household work history with the Arsenal (other choices were “I don’t really know enough about the arsenal to have an opinion” and “Overall, I believe the arsenal causes more harm than good in the New River Valley”). This finding is consistent with risk perception research showing that tangible benefits, such as employment, lower one’s perceived risk [21,22]. 

Those who expressed they do or might have concerns about the Arsenal were prompted to describe their primary concern. Of the 239 write-in responses to this open-ended question, 54% mentioned pollution/contamination or health of the environment. Another follow-up question specifically asked respondents to mark whether they had concerns related to: open-air burning; noise/vibrations; discharges into the New River; or occupational hazards for workers. “Discharges into the New River” and “occupational hazards” were the most frequently selected choices. Given that the pilot studies were related to open-air burning and not water contamination or occupational hazards, this question provided important insight into unresolved topics of community concern that should be addressed by future research. It is also important to note that while the study was initiated in response to community members’ environmental health concerns, the survey illuminated the fact that respondents with a household work history with the Arsenal commonly cited occupational hazards as a concern. 

#### 3.2.2. Interview Results

Despite outreach attempts to historically Black neighborhoods in the area, all but one of the interviewees in our sample were white, and one was white and American Indian. There were eight men and four women in the sample, and interviewees’ ages ranged from 31 to 70, with a median age of 45. Five interview respondents identified as liberal, progressive, or Democrat, five identified as conservative or Republican, two identified as independent, and one chose not to share their political views. All of the interviewees lived in the three zip codes adjacent to the RAAP site. 

When asked about the benefits the Arsenal provides to the community, interview respondents most often cited the jobs that RAAP has provided to the surrounding region and the role that it plays in national security. However, 10 out of 13 interview respondents also described specific concerns related to the Arsenal and other companies that occupy the property (one of which manufactures fireworks). Four respondents mentioned being disturbed by loud noises and explosions that occur without notice. As one interviewee noted, “Sometimes when the explosions are really big, they rock my house and upset my dog... Even when it’s thunder, your first thought is that something has happened at the Arsenal.” Another respondent echoed these concerns, stating, “Sometimes there will be strange explosions over there…Sometimes you’ll be outside and hear an explosion, and that’s a concerning issue. I wish there was a way to check to see if it is just the fireworks company doing testing or if it is an emergency.” Although accidents are rare at RAAP, when they do happen, they can leave a lasting impact on those nearby. One interviewee who grew up close to the Arsenal described how, when she was young, “every once in a while something would blow up, or they would have an accident… And you would find out later that people died.” Another interviewee noted that he knows a number of people who live close to the Arsenal “who believe it has affected their mental health, as far as stress and worry and paranoia.” 

The “stress and worry” noted above is not limited to explosions. The greatest concerns expressed by the respondents were associated with the possibility of environmental health consequences from open-air burning of waste materials from propellant manufacturing and water pollution from the facility. As one respondent stated, “We’ve had health-related things happen to us that we can’t directly attribute to the Arsenal, but…we often wonder if there is a connection. We can’t pinpoint it because there is not enough research or data to know.” Another interviewee wondered how far plumes from the open burning grounds travel and noted that she sometimes detects a “really strange chemical smell” when driving down Price’s Fork Road. Uncertainty was a strong theme; as one interviewee stated, “I feel like the longer that we live here, the more uncertainty we have about how it affects us. We feel like it has to have an effect on us at some level, which is why we are concerned.” The interview transcripts make clear that the perceived dearth of information about what is taking place at the Arsenal is central to community members’ concerns. As one interviewee articulated, “I have just been disturbed by the lack of transparency of the scientific information.” Another explained, “My understanding is they do lots of scary stuff there, and the question is, does that actually affect the community or does it not?...I’d like there to be testing to know what [the water discharges and open burning] does to the surrounding area.” Many of the respondents called for more monitoring, both of the air and the water, and for RAAP to share the results of such monitoring with the public. 

Some respondents observed that the communication between RAAP and the community had been getting better, and they expressed appreciation for those improvements. One interviewee recalled the first quarterly meeting she attended. After that meeting, she thought, “‘Whoa, they’re really up to bad stuff, and this is, you know, like a plague on our community.’” But then, as the meetings became more open and more information was shared with the community, that feeling began to change: “I think the improved question-and-answer sessions under the new Commander has…made me sort of back up and say, ‘You know, we just need more information’ to know whether we should really freak out or not.” This interviewee stated that she has come to believe that the Arsenal staff “genuinely want to engage” with the community. 

As the analysis of the interviews revealed, many respondents recognize and appreciate the Arsenal’s efforts at increased transparency taking place but would like to see those efforts go further. In particular, additional monitoring and more publicly available data on both air and water pollution are outcomes that these individuals would like to see. Interviewees requested continuous air and water monitoring of contaminants like particulate matter, dioxins, and furans, including at Belview Elementary School; monitoring exposure to contaminants via bio-indicators such as cow blood; and timely public access to data from monitoring.

#### 3.2.3. Student Integration of Pilot Study, Literature Review, Survey and Interview Results

Students felt that their literature review was limited by the paucity of recent public data about nearby well water, the absence of data about municipal water downstream of the Arsenal, and an absence of data about thyroid cancer below the county level. The class had discussed at length whether they felt confident making recommendations for next steps. They declined to speak for the community, instead suggesting that RAAP and local governments seek out community feedback to learn what additional measures community members wanted to see taken in the wake of the Virginia Tech research. Students did, however, acknowledge several likely areas of ongoing attention. First, they anticipated community interest in a systematic study of well water, given that a 2015 report by the Agency for Toxic Substances and Disease Registry [23] encouraged well owners to test periodically to ensure their drinking water is safe. Second, students recognized that if concerns persisted or increased regarding thyroid cancer despite the lower-than-average county incidence rates, residents might be interested in requesting a study of thyroid disease incidence at the zip code or neighborhood level. Third, students noted that the Arsenal’s increased responsiveness and provision of additional data had decreased anxiety and recommended that the Arsenal continue to work towards greater public accessibility of environmental data regarding emissions. In particular, students observed, community members want to know more about discharges into the New River (a 2018 report by the Virginia Department of Environmental Quality and Virginia Tech listed the concentrations of PCBs released from the arsenal as “varies”) and were eager for all monitoring reports to be made publicly accessible in a timely fashion. Finally, students suggested the implementation of a public alert system that would give advance warning and updates, including alerts about noise and vibrations experienced off site, as a means of further strengthening the community trust.

### 3.3. Sharing the Results with the Community 

The students highlighted key findings from their research in local public presentations before the Pulaski County Board of Supervisors and the Montgomery County Board of Supervisors, and at a RAAP quarterly meeting and a New River Valley Planning Commission work session. The students’ presentations were well attended and well received, potentially for reasons explored below in Discussion. 

In the spring 2019 semester, a team of five students and faculty continued the work of communicating the class’s findings regarding the Arsenal and environmental health by publishing a website, www.raap.fralin.vt.edu, in the hopes of reinforcing strides made towards transparency and dialogue.

## 4. Discussion

Our research team’s outreach to RAAP and the research undertaken in the Appalachian Community Research course resulted in collaborative efforts to improve our understanding of emissions from RAAP, residents’ perceptions of RAAP, and improving communication between researchers, residents, and students. It is possible that community concerns related to air pollution from RAAP might have been resolved (to the extent that they were resolved) without this class or its related projects. The Arsenal had already made a number of good-faith attempts at responding to the community prior to the Rural Environments Group’s involvement. In May 2017, RAAP switched its electricity generation to natural gas and closed its coal burning plant; future reports should show decreased particulate matter in the atmosphere as a result. In 2018, staff were exploring the possibility of a 5-acre solar array to provide 5–7% of the plant’s electric needs, as well as potentially using nitric acid wastewater as a biofuel. Improvements in propellant production included the removal of perchlorates and dinitrotoluene as ingredients, and staff were exploring removing lead from propellant ingredients with the cooperation of their end users. By the March 2018 quarterly meeting, RAAP announced that the funds for a new incinerator were forthcoming and provided a 34-month timeline for its completion with normal operation anticipated by 2023. By July 2018, RAAP had filed its air quality permit application for a new Energetic Waste Incinerator with DEQ. In addition, RAAP employees discovered a way to safely cut waste that typically went to the open burning grounds into smaller sections so that it could be sent to the existing incinerator instead, and RAAP leadership engaged regulatory agencies to allow the change. By the time of the air quality pilot study in the summer of 2018, RAAP had reduced the volume of waste it sent to the open burn ground by 42 percent and had reduced the amount of lead sent by 95 percent.

Although these positive changes were taken independently of our research team’s involvement, RAAP staff shared with us that our collaboration had important effects on their relationship with the community. Reflecting on what they gained from our collaboration, a RAAP representative stated in an email communication that our research “substantially impact[ed] our understanding of public perceptions…Overall, it is our opinion that the collaboration with VT was instrumental in defusing tensions [with the community] and creating an honest, productive dialogue based on facts and evidence rather than emotion.” In addition, this representative noted that RAAP could not have done the off-site air sampling “as quickly as we could with VT as a partner.” RAAP staff also invited Virginia Tech air engineers to provide assistance in selecting and siting new air monitoring equipment to be installed on site at the plant. Air monitor tests were conducted weekly during the second half of 2018 with plans to reassess frequency in January 2019, given that monitors were not detecting any levels of concern.

We believe that the Appalachian Community Research course was central to the success and positive reception of the research for multiple reasons. Student involvement seemed to have a disarming effect at multiple junctures. Virginia Tech has a mixed reputation in the New River Valley. Largely its presence is welcome, and residents cheer for the football team. Though many Southwest Virginians respect Virginia Tech’s stature as a research institution, others are critical of funding models that they believe skew researchers’ findings in favor of industry and the US Department of Defense, and still others are critical of research that they believe promotes more regulation and “big government”. Student involvement in the project may have appealed to both fans and critics. On the one hand, students are viewed as a nuisance (traffic and erratic driving, partying noise and mess). But residents also take an indulgent or paternalistic view of students, perhaps partly out of nostalgia for their youth or affection for their own children. Interviewees genuinely seemed to want to be helpful to students, perhaps finding them less threatening than other researchers. Elected officials were gracious and generous with their time in meeting with students to teach them about their localities and constituents. These officials were able to take on the role of educators, instead of simply being on the receiving end of recommendations made by academics. 

Boards of Supervisors likely felt less heat from constituents for cooperating with undergraduates than might have been the case had they worked with faculty alone. Perhaps residents perceived students as less likely to have selfish motives or conflicts of interest than grant-funded researchers, career-building faculty, the public relations-sensitive university, or Arsenal staff. As one commentator put it in response to a radio report announcing our study, Virginia Tech faculty researchers are perceived to “have a vested interest. I voiced as much at the last meeting. I questioned...why there are not other scientists from other research institutions involved” [24]. During the question and answer sessions after student presentations, Arsenal critics seemed less inclined to be vocally hostile to or suspicious of students compared to other purveyors of information, who are often accused of hiding portions of data from the public. During the interview stage, students were able to sincerely and compellingly invite community members to take on the role of teachers, and interviewees in turn may have become more invested and confident in student-presented findings. 

Through this project, students gained the skills and the confidence to engage the public and communicate data relevant to public health. They both embraced the “real world” element of the course but also recognized the pitfalls of wanting to be heroes. “I subconsciously wanted to find a scandal,” one wrote, “to expose some kind of injustice that the military was perpetrating against American citizens” (Environmental Policy and Planning major). Recognizing that impulse, and the ways it might affect researchers’ conclusions, was a key lesson. 

In their final reflection papers, students described the course as a rare opportunity to engage with Appalachian communities surrounding Virginia Tech. Students reported that they became “cognizant” that we, “as college students, hold a certain privilege over the community that surrounds us, and most of us aren’t even from the area.” A biochemistry major confessed: “I must admit, with some shame, that this project was the very first time that I have ever engaged with the people living in this area who are not associated with Virginia Tech.” The same student reported that their “research and experience in this class, first and foremost, taught me to center the community affected when tackling any type of issue, especially when it concerns public health.” Students praised the course for teaching them the importance of keeping community needs and perspectives central to academic research. An environmental science major observed that “the class was completely oriented around the comments we received from regular people. Without them, there would have been no class. In my experience, academia rarely seems to be more than peripherally connected with its greater community. This class was very special in the way that it was entirely shaped by” its community. A geography and sociology major reported that the class taught her that “the researcher role is more about learning from the community, rather than imbuing knowledge upon them.” She came to “understand just how important professionalism, compassion, and sincerity is in research because it shapes how much communities will trust you and value what you have to share with them and what information they are willing to share with you.”

Students’ final reflection papers also asserted the value of transdisciplinary collaboration. “It was exciting”, reported a criminology and sociology major, “to tackle a project that was so much bigger than myself” and to “be part of a team.” The environmental science student “loved having such a diverse team of professors not only at our disposal, but working alongside us.” An environmental policy and planning major “learned a lot about the differences in research methods in the social sciences from other disciplines.” The course taught a political science major “the value of intersecting scientific research, an environmental justice framework, and community engagement when working together with several actors to address a problem.”

The environmental policy and planning major expressed hope that “our efforts encourage further transparency and eas[y] access of information for community members and future researchers,” while the human development major expressed hope that “our research helps community members reach some clarity about what is happening now and what future steps need to be taken.” It indeed appears that the students’ research project may have contributed to improved lines of communication—and possibly trust—between RAAP and the surrounding communities. News coverage in the *Virginia Mercury* attests that many community members expressed increased confidence in RAAP and appreciation for RAAP’s transparency and communication. The county Boards of Supervisors reported expressions of gratitude from constituents who have long voiced concerns about the Arsenal. 

The Arsenal’s welcoming of our involvement was crucial to the success of the project, both because it allayed politicians’ concerns that they not be seen as conducting a witch hunt and because it was part of a larger commitment to responding to long-standing community tensions and demands for transparency. At the same time, it is important not to downplay the role of the “squeaky wheels” in the community. It was their persistent demands that prompted responsiveness and cooperation by both the Arsenal and county Boards of Supervisors, and which pointed the researchers towards areas of concern for scrutiny. While our research did not confirm those residents’ suspicions, we hope that our project reinforced residents’ sense of agency.

## 5. Conclusions

Scholars in different disciplines operate based on differing incentives, research costs, and teaching duties. We found that moving from multidisciplinary to transdisciplinary research depended upon deliberate institutional practices that facilitated and incentivized transdisciplinary research through providing resources and structures, such as funding for research teams and opportunities to co-teach small, interdisciplinary classes. Conducting our research in tandem with a community-engaged undergraduate research course was key to a collaborative production of knowledge that fused different disciplinary paradigms and epistemological orientations. Designing this research as we simultaneously developed the undergraduate curriculum facilitated our ability to foster a deeper understanding of and respect for different disciplinary strengths by researchers and students alike. Although various components of the project were not transdisciplinary (for instance, pilot studies were completed by individual lab groups), the class served as the site of synthesis, allowing both faculty and students to learn from the other disciplines represented. The role of the university–community partnership in shaping the class was crucial in the successful transition to transdisciplinarity for multiple reasons. Community needs provided a shared impetus for the research among faculty and highlighted differing disciplinary conventions regarding the appropriate role of local knowledge in relation to scholarly knowledge. Government agency funding that required a course informed by community partners lent legitimacy to the research and helped assure substantive community participation in setting the research agenda. Students’ interactions with residents served as a motivation for integrating and reconstituting available disciplinary knowledges into a cohesive whole. 

In conclusion, institutional support yielded substantial benefits in terms of affirming community engagement, forging new relationships among institutions and among faculty members, enriching students’ intellectual and vocational growth, as well as contributing to the land-grant mission of Virginia Tech and helping to fulfill the university’s motto: *Ut Prosim*, “That I May Serve”. Our results suggest that institutional structures and initiatives which support co-instruction of small, research-based courses, in addition to seed funding for interdisciplinary studies, may help to further facilitate growth of transdisciplinary research capable of addressing complex problems that matter to broad constituencies.
